# Non-pharmacological pain relief in dialysis: commentary on a self-controlled study

**DOI:** 10.1177/17449871251370464

**Published:** 2025-09-17

**Authors:** Pernilla Garmy

**Affiliations:** Professor, RN, Kristianstad University, Sweden

Commentary on “Effect of cold needle application on the arteriovenous fistula cannulation-related pain” (Ozen et al 2025)

In their paper, Ozen et al. (2025) present a self-controlled, double-blind study exploring the effect of cold needle application on arteriovenous fistula (AVF) cannulation-related pain in haemodialysis patients. Thirty-three patients were included and served as their own controls: during the first three dialysis sessions, room-temperature needles were used; during the subsequent three sessions, needles cooled to 0–2°C were used. Importantly, both the patients and the nurses were blinded to the needle condition, as the nurses handled only the plastic hub, not the metal shaft.

The results were clear: cold needle application significantly reduced perceived pain compared to room-temperature needles. This simple, low-cost intervention holds promise for improving the patient experience in dialysis care.

Having worked with children receiving dialysis in a paediatric hospital, I have seen first-hand how the experience of needle cannulation varies greatly from child to child. Dialysis is not a one-time event, but it is a recurring, life-altering treatment that takes place several times a week, often over many years. The physical and emotional toll on both the patient and their family is substantial.

Although a single injection – such as a vaccination – may not seem to warrant extensive pain management, the cumulative effect of hundreds of cannulations is a different matter entirely.

That is why I found the findings of Ozen et al. (2025) so compelling. The intervention is not only effective but also simple, inexpensive and easily implemented in clinical practice. It has the potential to meaningfully reduce pain with no need for pharmacological agents or complicated equipment.

Similar conclusions were reached in a recent systematic review by Zukhra and Wanda (2024), which found that non-pharmacological methods – such as cold (cryotherapy), visual distraction, and aromatherapy – are effective in managing cannulation pain in children undergoing haemodialysis. Their work reinforces the idea that small, thoughtful adjustments in care can make a significant difference.

Future research should explore whether the pain-reducing effect of cold needle application is sustained over time, or whether repeated use leads to habituation and a return to baseline pain levels. It would also be of interest to examine whether this intervention has similar benefits in other clinical contexts where needles are frequently used.
